# Complete Genome Sequence of Altererythrobacter sp. Strain B11, an Aromatic Monomer-Degrading Bacterium, Isolated from Deep-Sea Sediment under the Seabed off Kashima, Japan

**DOI:** 10.1128/genomeA.00200-18

**Published:** 2018-03-22

**Authors:** Allyn H. Maeda, Shinro Nishi, Shun'ichi Ishii, Yasuhiro Shimane, Hideki Kobayashi, Junko Ichikawa, Kanako Kurosawa, Wataru Arai, Hideto Takami, Yukari Ohta

**Affiliations:** a Research and Development (R&D) Center for Marine Bioscience, Japan Agency for Marine-Earth Science and Technology (JAMSTEC), Yokosuka, Kanagawa, Japan; b R&D Center for Submarine Resources, JAMSTEC, Nankoku, Kochi, Japan; c R&D Center for Submarine Resources, JAMSTEC, Yokohama, Kanagawa, Japan

## Abstract

Altererythrobacter sp. strain B11 is an aromatic monomer-degrading bacterium newly isolated from sediment under the seabed off Kashima, Japan, at a depth of 2,100 m. Here, we report the complete nucleotide sequence of the genome of strain B11.

## GENOME ANNOUNCEMENT

Altererythrobacter is one of the genera within Alphaproteobacteria proposed by Kwon et al. (1). Various species belonging to this genus have been frequently isolated from marine environments, including sediments, seawater, and tidal flats (2). Several physiological studies have reported that Altererythrobacter strains possess degrading activity against recalcitrant organic hydrocarbons, such as alkanes (3) and polyaromatic hydrocarbons (4, 5), derived from petroleum. In addition, the potential genes responsible for alkane and benzo[*a*]pyrene degradation have also been found bioinformatically (3, 5). However, there is little information about the other aromatic compounds that naturally occur in ubiquitous plant biomasses (6). In this study, we successfully isolated a new type of Altererythrobacter strain from the marine sediment recovered from about 9 m under the seabed off Kashima, Japan (36.07° N, 141.79° E), at a depth of 2,100 m. The strain B11 can degrade aromatic monomers, such as *p*-coumaric acid, ferulic acid, and 4-hydroxybenzoic acid, which are components of various plant cell walls (7). Because the 16S rRNA gene sequence of strain B11 showed 98.1% identity with that of Altererythrobacter atlanticus 26DY36^T^ (8, 9), we designated it Altererythrobacter sp. strain B11.

Total genomic DNA of strain B11 was extracted using a NucleoSpin Plant II midikit (TaKaRa Bio) according to the manufacturer’s protocol. Whole-genome sequencing of strain B11 was performed by means of both Pacific Biosciences RS II (10) and Illumina HiSeq 2500 sequencers. A total of 126,732 PacBio reads (1,111,616,349 bases) were obtained using SMRT Analysis (v 2.3.0) and assembled into a contig (redundancy of 227-fold) with the Hierarchical Genome Assembly Process v 3 (HGAP3) assembler (11). Paired-end Illumina reads (2 × 101 bp, 24,220,470 reads) were used to correct the contig derived from the PacBio sequence (redundancy of 625-fold) to complete genome sequencing using the read-mapping program in CLC Genomics Workbench v 9 (CLC bio, Aarhus, Denmark).

The genome of strain B11 is composed of a single circular chromosome (3,842,046 bases), with a mean G+C content of 65.4%. We identified 3,645 protein-coding sequences (CDSs), 51 tRNAs, and 6 rRNAs by means of the MetaGeneAnnotator (12), tRNAscan-SE 1.23 (13), and RNAmmer 1.2 (14) servers, respectively. We manually annotated the predicted CDSs through an NCBI BLAST search and orthologous analysis using Kyoto Encyclopedia of Genes and Genomes (KEGG) orthology and NCBI Clusters of Orthologous Groups of proteins (COGs) as the protein databases.

We predicted overall metabolic and physiological functions of strain B11 using the metabolic and physiological potential evaluator (MAPLE) with bidirectional best-hit matches (15, 16). In addition, we successfully identified the genes encoding key enzymes responsible for the degradation of various aromatic compounds (17), such as multiple protocatechuate 3,4-dioxygenases and a 4-hydroxybenzoate 3-monooxygenase, in the genome. The genomic information of the newly isolated strain B11 will facilitate a better understanding of the metabolism for degrading recalcitrant aromatic compounds by the Altererythrobacter species in marine environments.

### Accession number(s).

This whole-genome shotgun project has been deposited in DDBJ/ENA/GenBank under the accession no. AP018498.
